# Co-occurrence of angioimmunoblastic T-cell lymphoma and aggressive-refractory plasma-cell neoplasm: Two new cases and literature review

**DOI:** 10.46989/001c.158183

**Published:** 2026-03-11

**Authors:** M. Christina Cox, Claudia Seimonte, Erica Giacobbi, Gianmario Pasqualone, Livio Pupo, Annagiulia Zizzari, Luca Franceschini, Adriano Venditti, Massimiliano Postorino

**Affiliations:** 1 Hematology Unit, Fondazione Policlinico Tor Vergata, Rome, Italy; 2 University of Rome Tor VergatDepartment of Biomedicine and Prevention, University of Rome, Tor Vergata, Rome, Italy; 3 Pathology Unit, Fondazione Policlinico Tor Vergata, Rome, Italy; 4 Department of Biomedicine and Prevention, University of Rome, Tor Vergata, Rome, Italy; 5 University of Rome Tor VergatHematology Unit, Fondazione Policlinico Tor Vergata, Rome, Italy

**Keywords:** T-cell lymphoma, Myeloma, Paraprotein, IgA, Angioimmunoblastic, refractory

## Abstract

Angioimmunoblastic T-cell lymphoma (AITL) is a rare and aggressive peripheral T-cell lymphoma of T-follicular helper (TFH) cell origin, characterized by systemic manifestations, immune dysregulation, and frequent secondary B-cell proliferations. While Epstein–Barr virus (EBV)-related diffuse large B-cell lymphoma develops in up to 20% of AITL cases, the occurrence of overt plasma cell neoplasms such as multiple myeloma (MM) has been documented only in isolated reports. We describe two new patients with AITL complicated by highly aggressive, treatment-refractory plasma-cell neoplasia, including the first documented case of primary cutaneous myeloma, and provide a comprehensive review of all published cases of concurrent systemic T-cell lymphoma (TCL) and plasma-cell neoplasm. A systematic search of PubMed, Web of Science, and Google Scholar identified 16 previously reported cases, yielding a total of 18 patients. These included several TCL subtypes. In most cases, the plasma-cell neoplasm occurred synchronously or shortly after TCL diagnosis. Approximately 50% of patients had an IgA paraprotein. Overall outcomes were dismal, with a median survival of less than two months following diagnosis of the second malignancy. EBV was detected in neoplastic plasma cells in only a minority of cases, suggesting alternative pathogenetic mechanisms such as cytokine-driven B-cell activation or shared clonal origin.

## Introduction

Angioimmunoblastic T-cell lymphoma (AITL) is a rare subtype of peripheral T-cell lymphoma derived from T-follicular helper (TFH) cells, as recognized in the 2022 World Health Organization (WHO) Classification of Hematopoietic and Lymphoid Neoplasms. Clinically, AITL is characterized by systemic manifestations, including fever, lymphadenopathy, rash, and immune dysregulation. Up to 30% of patients develop hypergammaglobulinemia, attributed to excessive cytokine secretion by neoplastic TFH cells, which promotes polyclonal B-cell activation and plasma cell differentiation.^1^

Notably, approximately 20% of AITL cases are associated with a concurrent Epstein–Barr virus (EBV)-driven diffuse large B-cell lymphoma (DLBCL), reflecting the profound immune dysregulation characteristic of this entity.^2^ In contrast, the development of overt plasma cell neoplasms, such as multiple myeloma (MM), is exceedingly rare and has been reported only in isolated cases (Table 1).

**Table 1.**
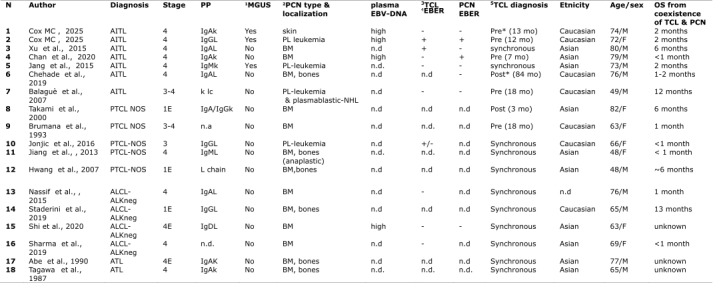
Clinical features of 18 patients with the coexistence of T-cell lymphoma and Plasma-cell Neoplasia **Footnote:**^1^MGUS: monoclonal gammopathy of uncertain significance; ^2^PCN: plasma-cell neoplasia; ^3^TCL: T-cell lymphoma; ^4^EBER: Epstein–Barr virus–encoded small RNAs ; ^5^TCL diagnosis: the diagnosis of TCL was carried out previous, synchronous or post the outbreak of plasma-cell neoplasm

Here, we describe two new patients with AITL complicated by treatment-refractory plasma cell neoplasia, including the first documented instance of primary cutaneous myeloma. We also provide the first comprehensive review of 16 previously published cases with concurrent systemic T-cell lymphoma (TCL) and plasma cell neoplasia (Table 1).

### Case Reports

#### Case 1

A 73-year-old man was admitted in March 2019 with fever, rash, generalized lymphadenopathy, syndrome of inappropriate antidiuretic hormone secretion (SIADH), dry cough, and profound fatigue. Laboratory investigations revealed moderate pancytopenia (neutrophils 0.86×10⁹/L, lymphocytes 0.56×10⁹/L, hemoglobin 9.3 g/dL, platelets 35×10⁹/L) and markedly elevated inflammatory markers (CRP 80 mg/L, ESR 100 mm/h, ferritin 2070 µg/L, LDH 520 U/L, and fibrinogen 580 mg/dL). Serum protein electrophoresis identified a small IgA-kappa paraprotein in the context of marked polyclonal hypergammaglobulinemia (γ-globulin ~40 g/L) and hypoalbuminemia (23 g/L).

Blood and urine cultures, serologies for HBV, HCV, and HIV were negative, but plasma EBV-DNA was elevated at 74,150 copies/mL. Empiric antibiotic therapy was ineffective. Bone marrow aspiration demonstrated trilineage cytopenia with mild dysplastic changes and 10–15% reactive, polytypic plasma cells. PET/CT revealed FDG-avid supradiaphragmatic lymph nodes (up to 2.5 cm) and small bilateral pulmonary nodules. Excisional lymph node biopsy confirmed AITL. Neoplastic T cells expressed CD2, CD3, CD4, CD5, CD10, PD-1, BCL6, CXCL13, and CD30 (10%), were negative for EBER, and had a Ki-67 proliferation index of approximately 40%.

The patient began Cyclophosphamide-Prednisone-Doxorubicin-Vincristine (CHOP) chemotherapy in April 2019, achieving complete remission of systemic symptoms, regression of lymphadenopathy, normalization of blood counts, and decreased gammaglobulin levels (25 g/L). After four cycles, he was re-admitted with fever, severe thrombocytopenia, and epileptic seizures. PET/CT showed only two small residual FDG-avid lung nodules. Bone marrow, brain MRI, and cerebrospinal fluid were unremarkable. High-dose dexamethasone (16 mg/day for several days) led to full recovery and correction of cytopenias.

Brentuximab vedotin was initiated in August 2019, but after three cycles, anemia and thrombocytopenia recurred. In October 2019, treatment was switched to lenalidomide with weekly dexamethasone. By January 2020, rash, fatigue, and pancytopenia reappeared, although PET/CT showed only a single small FDG-avid pulmonary lesion.

**Figure 1.**
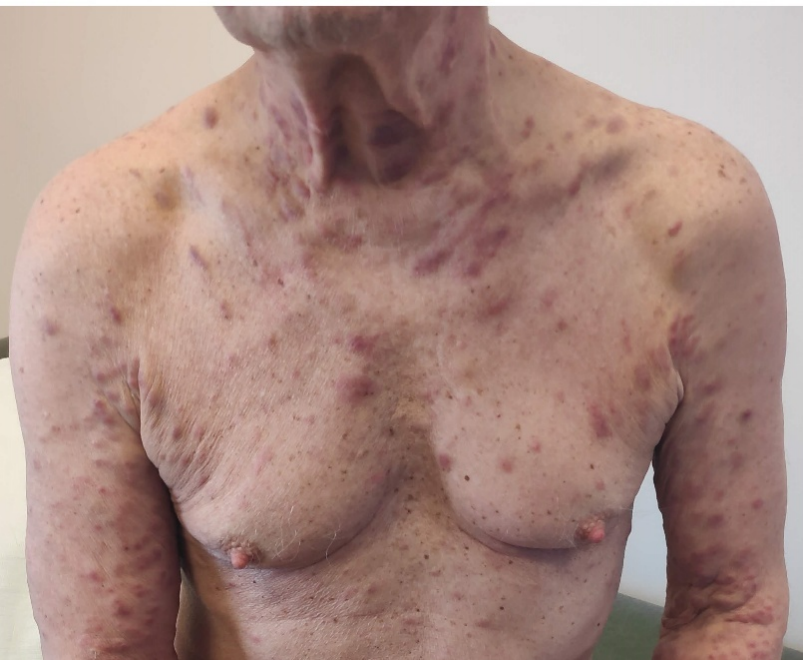
multiple skin lesions in primary cutaneous myeloma

In April 2020, new erythematous plaques developed on the trunk, neck, and upper limbs (Figure 1), coinciding with a sharp increase in the IgA-kappa paraprotein level (35 g/L) and a free light chain ratio >100. EBV-DNA was 68,280 copies/mL. Skin biopsy revealed a dermal infiltrate of clonal plasma cells (CD38+, CD138+, MUM1+, CD56±, IgA-kappa+) consistent with cutaneous myeloma. FISH demonstrated TP53 deletion; Epstein-Barr virus-encoded small RNA (EBER was negative in plasma-cells. Repeat bone marrow biopsy showed no evidence of AITL or myeloma. A new PET/CT scan showed persistence of FDG uptake in the small lung nodule, while no FDG lesions were detected in the bones and other organs. The patient received pomalidomide, bortezomib, and dexamethasone, but the disease rapidly progressed. He developed acute renal failure and died of sepsis in June 2020.

#### Case 2

In May 2021, a 71-year-old woman presented with night sweats, low-grade fever, and generalized lymphadenopathy. CT imaging revealed multiple enlarged lymph nodes and hepatosplenomegaly. An initial lymph node biopsy showed only reactive changes.

She was referred to our center in June 2021 for further evaluation. Her history included nodular goiter (euthyroid), celiac disease, and resected dermatofibrosarcoma (2016). Laboratory studies showed anemia (hemoglobin 94 g/L), thrombocytopenia (83×10⁹/L), normal leukocytes (5.45×10⁹/L; neutrophils 62%, lymphocytes 30%, monocytes 8%), elevated LDH (350 U/L), and β₂-microglobulin (11.4 mg/L). Serum immunoglobulins (Ig) revealed marked polyclonal hypergammaglobulinemia with elevated IgA and IgG levels. Serology showed isolated anti-HBc positivity, consistent with past HBV infection.

Bone marrow examination demonstrated a 10% infiltrate of CD3+ T cells and 20% polytypic CD138+ plasma cells, with scattered EBV-positive cells by EBER in situ hybridization. Peripheral blood flow cytometry was non-diagnostic. Plasma EBV PCR was 247,000 copies/mL. PET/CT (August 2021) demonstrated hypermetabolic lymphadenopathy and splenic involvement (SUVmax 7.4). An excisional axillary lymph node biopsy confirmed EBV-positive AITL (CD2+, CD3+, CD5+, CD4+, PD-1+, EBER+) coexisting with EBV-positive DLBCL (CD20+, CD79a+, CD10+, EBER+), with a Ki-67 index of 75%. The composite lymphoma was staged as III-B.

Given her age and dual pathology, R-GEMOX chemotherapy (rituximab, gemcitabine, oxaliplatin) was initiated in September 2021. After one cycle, EBV-DNA became undetectable, but following the second cycle, she developed *Staphylococcus aureus* sepsis and *Escherichia coli* urinary infection, with EBV reactivation (72,800 copies/mL). PET/CT showed persistent lymphadenopathy, new bone lesions, ascites, and pleural effusion. Therapy was held until recovery.

In January 2022, lymph node biopsy confirmed persistent AITL. Rituximab-Cyclophosphamide-Liposomal Doxorubicin-Prednisolone-Vincristine therapy was commenced, leading to gradual clinical improvement and a decline in EBV viremia (7860 cp/mL). She completed six cycles by April 2022 without major toxicities. In April, after four cycles of RGEMOX, she achieved complete remission by PET-CT scan, EBV viremia was 3780 cp/mL, hemoglobin and platelets normalized, although a mild lymphocytosis (~5×10⁹/L) was noted.

In May 2022, she experienced an abrupt clinical decline with severe anemia (Hb 8.6 g/dL) and extreme leukocytosis (138.5×10⁹/L). Peripheral blood smear and flow cytometry showed 77% circulating plasma cells (CD38+, CD138+, CD56±, CD20±, CD19−), diagnostic of primary plasma cell leukemia (pPCL). Serum studies demonstrated an IgG-lambda monoclonal protein, FLC λ 15.11 mg/dL, FLC κ 6.19 mg/dL (κ/λ ratio 0.41), and λ Bence Jones proteinuria. β₂-microglobulin was 10.8 mg/L, albumin 3.6 g/dL, renal function and calcemic level were within normal ranges (ISS stage III). LDH was 7500, and EBV peaked at 4.977.500 cp/mL, but plasmablasts were not analyzed for EBV-DNA integration. She was hospitalized and treated with plasmapheresis, high-dose dexamethasone, and bortezomib, but her white blood cell count continued to rise, peaking at 203×10⁹/L. Despite aggressive management, she developed septic shock and died shortly thereafter.

## Discussion

We report two new patients with concurrent angioimmunoblastic T-cell lymphoma (AITL) and plasma cell neoplasia, along with the first comprehensive review of similar cases published to date. One of our patients represents the first documented case of primary cutaneous myeloma (Figure 1). In fact, cutaneous involvement by myeloma is exceedingly rare (1–2%), and typically occurs in advanced-stage disease as a secondary localization.^3^ The second patient developed a primary plasma-cell leukemia. In both individuals, the plasma-cell dyscrasia followed an aggressive, treatment-refractory course, leading to death within two months from diagnosis of both malignancies.

T-cell lymphomas (TCLs) and multiple myeloma (MM) originate from distinct lymphoid lineages, and their coexistence in a single patient has been only anecdotally reported. To date, 18 cases—including the two described herein—have been documented. Their median age (70.5 years) and male-to-female ratio did not differ from those typically reported in TCL. These cases involved several TCL subtypes (Table 1): seven AITL, five peripheral T-cell lymphomas not otherwise specified (PTCL-NOS), four ALK-negative anaplastic large-cell lymphomas (ALCL), and two adult T-cell leukemia/lymphomas (ATL). This finding was rather unexpected, as plasma-cell proliferations are typically associated with lymphomas derived from follicular helper TFH cells. Indeed, most TCL subtypes secrete cytokines that profoundly disrupt immune regulation and may promote the uncontrolled proliferation of other immune cell populations.^4–6^

Similarly, in T-cell large granular lymphocytic leukemia (T-LGLL)—a chronic T-cell neoplasm—associations with monoclonal gammopathy of undetermined significance (MGUS) is frequently observed, and even MM has been reported. However, in contrast to TCLs, both T-LGLL and plasma-cell dyscrasias usually follow an indolent course.^7–9^

In most cases of this series, the plasma-cell neoplasia was diagnosed synchronously or a few months after TCL (Table 1), suggesting that their co-occurrence is unlikely to be coincidental. Interestingly, the distribution of monoclonal protein isotypes in these patients differs from that in typical MM: IgA was the most frequent (~50%), followed by IgG (~17%), IgM and light-chain types (~12% each), and IgD (5%). Four out of 18 patients developed primary plasma-cell leukemia, and in five who had FISH analysis of plasma-cells, this showed high-risk cytogenetic aberrations, including TP53 deletion in four of them (Table S1). The overall outcomes were poor, as the median survival after diagnosis of the second malignancy was less than two months (range, 1–13 months). Deaths were mainly attributable to rapid disease progression, refractoriness to therapy, and infectious complications.

Several mechanisms may concur with the development of plasma-cell neoplasm in TCL:

1. Profound immunosuppression in TCLs, particularly AITL, facilitates Epstein–Barr virus (EBV) reactivation in B cells. In fact, most patients exhibit high plasmatic titers of EBV-DNA, which typically decline during remission, as in the two new cases herein reported. EBV-transformed B-cell clones may undergo malignant transformation; indeed, at diagnosis, up to 20% of AITL cases harbor a concurrent EBV-related diffuse large B-cell lymphoma (DLBCL).^2^ Conversely, in our series, EBV was detected in neoplastic plasma cells in only a minority of cases, suggesting that alternative pathogenic mechanisms may be predominant in the concurrent development of TCL and MM. Indeed, EBV-negative DLBCLs with marked plasmocytic differentiation—ranging from mature plasma cell proliferations to plasmolysis lymphoma—have also been described in AITL.^10^ However, EBV reactivation induces immune dysregulation, potentially contributing to the development of MM.^11^

2. Immune stimulation driven by cytokine dysregulation from neoplastic T cells^4,6^ — particularly those of TFH origin — and sustained antigenic stimulation within the tumor microenvironment may promote aberrant B-cell activation and plasma cell differentiation.^5^ In fact, anecdotal cases of extreme polyclonal plasmacytosis have also been described in AITL.^12,13^ Several cytokines secreted by malignant T cells, including IL-5, IL-6, IL-21, and TGF-β1, can promote B-cell survival, differentiation, and class switching—most notably toward IgA production.^14,15^ This cytokine milieu may account for the high frequency of IgA-type MM observed in this series. (Table 1).

3. Direct cellular interactions between neoplastic T cells and plasma cells, which have been occasionally observed histologically. Magro et al. reported that both AITL and cutaneous T-cell lymphomas may be infiltrated by clonal plasma cells.^16,17^ Such close cell-to-cell interactions could promote reciprocal growth of both tumor populations. Indeed, plasmablasts produce high amounts of IL-6, which is also pivotal for inducing the TFH differentiation program^18^

4. A common hematopoietic progenitor that acquires distinct oncogenic mutations may lead to divergent clonal evolution. In fact, AITL tumorigenesis is a multistep process, and premalignant cells may also differentiate into tumor-infiltrating B cells that ultimately mature into Ig-secreting plasma cells. Indeed, Fujisawa and collaborators^19^ showed that TET2 and DNMT3A mutations were present in both T-lineage tumor cells and infiltrating clonal B cells in the majority of analyzed patients.

5. A lineage switch may also be hypothesized.^20^ In fact, rearrangements of Ig genes as well as T-cell receptor genes have been reported in up to 40% of AITL samples.^21^ Unfortunately, in the two cases here described, TCR and Ig gene rearrangements were not assessed in either the MM or the T-lymphoma cells. However, lineage shift or a common progenitor origin was ruled out by molecular analyses in a few cases from this series.^17,22^

6. Previous therapies—including corticosteroids, immunomodulatory agents, and cytotoxic drugs—may also alter immune surveillance and the bone marrow microenvironment, predisposing to secondary neoplasms.

Further studies are needed to elucidate the biological mechanisms underlying the co-occurrence of TCL and MM, which may be underreported and associated with a dismal prognosis. Indeed, such patients should be considered for alternative or targeted therapies, such as anti-interleukin-6 antibodies, to disrupt potential synergism between neoplastic T cells and neoplastic plasma cells. Finally, we recommend that patients with TCL who present with hypergammaglobulinemia or paraproteinemia be thoroughly assessed and longitudinally monitored to rule out this rare but clinically significant association.

### Authors’ Contribution

Conceptualization MCC

Data curation CS, EG, GMP

Formal Analysis MCC, CS

Funding acquisition

Supervision MP, AV

Writing – original draft CS, GMP

Writing – review & editing MCC, CS, EG, GMP, AZ, LP, LF, MP, AV

### Competition of Interest – COPE

None of the Authors has competing interests to be disclosed

### Informed Consent Statement and Ethics approval

Informed consent was obtained from both patients. All authors and institutions have approved this manuscript for publication. Our institution does not require ethical approval for case series.

## Supplementary Material

Supplemental Table 1

## Data Availability

All are available upon reasonable request.

## References

[1] Advani R. H., Skrypets T., Civallero M., Spinner M. A., Manni M., Seog Kim W., Shustov A. R., Horwitz S. M., Hitz F., Elena Cabrera M., Dlouhy I., Vassallo J., Pileri S. A., Inghirami G., Montoto S., Vitolo U., Radford J., Vose J. M., Federico M. (2021). Outcomes and prognostic factors in angioimmunoblastic T-cell lymphoma: final report from the international T-cell Project. http://ashpublications.org/blood/article-pdf/138/3/213/1814765/bloodbld2020010387.pdf.

[2] Zettl A., Lee S.-S., Rüdiger T., Starostik P., Marino M., Kirchner T., Ott M., Konrad Müller-Hermelink H., Ott G. (2002). Am J Clin Pathol.

[3] Jurczyszyn A., Olszewska-Szopa M., Hungria V., Crusoe E., Pika T., Delforge M., Leleu X., Rasche L., Nooka A. K., Druzd-Sitek A., Walewski J., Davila J., Caers J., Maisnar V., Gertz M., Gentile M., Fantl D., Mele G., Vesole D. H., Castillo J. J.. (2016). Cutaneous involvement in multiple myeloma: a multi-institutional retrospective study of 53 patients. Leukemia and Lymphoma.

[4] Gupta M., Stenson M., O'Byrne M., Maurer M. J., Habermann T., Cerhan J. R., Weiner G. W., Witzig T. E. (2016). Comprehensive serum cytokine analysis identifies IL-1RA and soluble IL-2Rα as predictors of event-free survival in T-cell lymphoma. Annals of Oncology.

[5] Yadav M., Uikey B. N., Rathore S. S., Gupta P., Kashyap D., Kumar C., Shukla D., Vijayamahantesh Chandel, Chandel A. S., Ahirwar B., Singh A. K., Suman S. S., Priyadarshi A., Amit A. (2023). Frontiers in Oncology.

[6] Yi J. H., Ryu K. J., Ko Y. H., Kim W. S., Kim S. J. (2019). Profiles of serum cytokines and their clinical implications in patients with peripheral T-cell lymphoma. Cytokine.

[7] Pelliccia S., Di Napoli A., Naso V., Alma E., Rebecchini C., Cox M. C. (2013). Very long-lasting remission of refractory T-large granular lymphocytes leukemia and myeloma by lenalidomide treatment. European Journal of Haematology.

[8] Braunstein Z., McLaughlin E., Ruiz M., Wei L., Bumma N., Benson D., Devarakonda S., Chaudhry M., Khan A., Cottini F., Hanel W., Baiocchi R., Chung C., Addison D., Couette N., Meara A., Jarjour W., Porcu P., Mishra A., Reneau J.C., Rosko A.E., Brammer J.E. Incidence, Treatment, and Survival of Patients With T-Cell Lymphoma, T-Cell Large Granular Leukemia, and Concomitant Plasma Cell Dyscrasias. Front Oncol.

[9] Sidiqi M. H., Aljama M. A., Viswanatha D. S., Dingli D. (2019). T-cell large granular lymphocytic leukemia and plasma cell disorders. Haematologica.

[10] Balague´ O., Martı´nezmartı´nez A., Luı´s Colomo L., Rosello´ E. R., Garcia A., Martı´nezmartı´nez-Bernal M., Palacı´n A., Fu K., Weisenburger D., Colomer D., Burke J. S., Warnke R. A., Campo E. (2007). Epstein-Barr Virus Negative Clonal Plasma Cell Proliferations and Lymphomas in Peripheral T-cell Lymphomas A Phenomenon With Distinctive Clinicopathologic Features.

[11] Li J., Tan R., Yang B., Du C., Tian J., Yang Z., Tang D. (2025). Genetic evidence identifies a causal relationship between EBV infection and multiple myeloma risk. Scientific Reports.

[12] Adachi Y., Hino T., Ohsawa M., Ueki K., Murao T., Li M., Cui Y., Okigaki M., Ito M., Ikehara S. (2015). A case of CD10-negative angioimmunoblastic T cell lymphoma with leukemic change and increased plasma cells mimicking plasma cell leukemia: A case report. Oncology Letters.

[13] Mejia Saldarriaga M., Alhomoud M., Roboz G., Allan J. N., Ruan J., Ouseph M. M., Simonson P. D., Bustoros M., Niesvizky R. (2023). Angioimmunoblastic T-cell lymphoma presenting with severe plasmacytosis mimicking plasma cell leukemia. American Journal of Hematology.

[14] Dullaers M., Li D., Xue Y., Ni L., Gayet I., Morita R., Ueno H., Palucka K. A., Banchereau J., Oh S. K. (2009). A T Cell-Dependent Mechanism for the Induction of Human Mucosal Homing Immunoglobulin A-Secreting Plasmablasts. Immunity.

[15] Tokunaga T., Shimada K., Yamamoto K., Chihara D., Ichihashi T., Oshima R., Tanimoto M., Iwasaki T., Isoda A., Sakai A., Kobayashi H., Kitamura K., Matsue K., Taniwaki M., Tamashima S., Saburi Y., Masunari T., Naoe T., Nakamura S., Kinoshita T. (2012). Retrospective analysis of prognostic factors for angioimmunoblastic T-cell lymphoma: a multicenter cooperative study in Japan. https://doi.org/10.1182/blood-2011-08.

[16] Magro C. M., Ruan J., Grossman M., Hedayat A. A. (2019). Monoclonal plasma cell infiltrates in the setting of cutaneous follicular helper T cell lymphoproliferative disorders. Annals of Diagnostic Pathology.

[17] Suárez A. E., Artiga M. J., Santonja C., Montes-Moreno S., De Pablo P., Requena L., Piris M. A., Rodríguez-Pinilla S. M. (2016). Angioimmunoblastic T-cell lymphoma with a clonal plasma cell proliferation that underwent immunoglobulin isotype switch in the skin, coinciding with cutaneous disease progression. Journal of Cutaneous Pathology.

[18] Chavele K.-M., Merry E., Ehrenstein M. R. (2015). Cutting Edge: Circulating Plasmablasts Induce the Differentiation of Human T Follicular Helper Cells via IL-6 Production. The Journal of Immunology.

[19] Fujisawa M., Chiba S., Sakata-Yanagimoto M. (2017). Recent Progress in the Understanding of Angioimmunoblastic T-cell Lymphoma. J Clin Exp Hematop.

[20] Péricart S., Waysse C., Siegfried A., Struski S., Delabesse E., Laurent C., Evrard S. (2020). Subsequent development of histiocytic sarcoma and follicular lymphoma: cytogenetics and next-generation sequencing analyses provide evidence for transdifferentiation of early common lymphoid precursor—a case report and review of literature. Virchows Archiv.

[21] de Leval L., Gisselbrecht C., Gaulard P. (2010). Advances in the understanding and management of angioimmunoblastic T-cell lymphoma. Br J Haematol.

[22] Shi X., Wu J., Jiang Q., Zhang S., Chen W., Yu X., Liu Y., Chen M., Peng J., Li T., Zhu Y., Xi X. (2020). Synchronous diagnosis of anaplastic large cell lymphoma and multiple myeloma in a patient: A case report. Medicine (Baltimore).

