# Supplementary material for: Co-occurrence of angioimmunoblastic T-cell lymphoma and aggressive-refractory plasma-cell neoplasm: Two new cases and literature review
Source: Clin Hematol Int. 2026 Mar 11;8(1):49–55. doi: 10.46989/001c.158183 (PMC12991438; doi:10.46989/001c.158183)
Supplement: Supplemental Table 1 [file chi_2026_8_1_158183_332316.docx]

**Co-occurrence of Angioimmunoblastic T-cell Lymphoma and Aggressive-Refractory Plasma-cell Neoplasm: Two New Cases and Literature Review**

**Maria Christina Cox^1^, Claudia Seimonte^2^, Erica Giacobbi^3^, Gian Mario Pasqualone^2^, Livio Pupo1, Annagiulia Zizzari^1^, Luca Franceschini^1^, Adriano Venditti^2^ and Massimiliano Postorino^2^.**

**1 Hematology Unit, Fondazione Policlinico Tor Vergata, Rome, Italy**

**2 Department of Biomedicine and Prevention, University of Rome, Tor Vergata**

**3 Pathology Unit, Fondazione Policlinico Tor Vergata, Rome, Italy**

**Corresponding Author: Maria Christina Cox, email** [**chrisscox@gmail.com**](mailto:chrisscox@gmail.com)

**Hematology, Policlinico Tor Vergata, Rome 00133, Italy**

Supplementary Table 1: Genetic abnormalities in neoplastic plasma-cells of five patients who had coexisting T-cell lymphoma.

| Author | T-cell Lymphoma | Neoplastic plasma-cell |
| --- | --- | --- |
| Shi et al., 2020 | DUSP22  rearrangement with t (6p25) (IRF4/DUSP22) | FISH results of del (17) (p13) (P53/CEP17), t (14;20) (q32;q12) (IGH/MAFB), t (14;16) (q32;q23)  (IGH/MAF), and t (4;14) (p16;q32) (IGH/FGRF3), |
| Staderini et al. 2019 |  | *t*(14;16) in 100% and  del17p13 in 64% of the cells analyzed. No TCR rearrangement |
| Cox et al. 2025 |  | 17p (del(17p13), *t*(14;16) (q32;q12) (IGH/MAFB), |
| Jiang et al. 2013 |  | Complex |
| Chedade et al. 2019 |  | 17p (del(17p13), +high-risk abnormalities |
